# Shifting social norms, behavioural intentions and opposition to female genital mutilation: Effectiveness of layered community interventions in a high prevalence setting in Kenya

**DOI:** 10.1371/journal.pgph.0007044

**Published:** 2026-09-10

**Authors:** Dennis Juma Matanda, Martina Mchenga, Stella Muthuri, Francis Obare, Teresa Awili, Zamzam Hassan, Chi-Chi Undie

**Affiliations:** 1 Population Council-Kenya, Nairobi, Kenya; 2 Independent Researcher, Johannesburg, South Africa; 3 Population Council, Inc., Nairobi, Kenya; 4 ActionAid International Kenya, Nairobi, Kenya; University of Nottingham School of Medicine, UNITED KINGDOM OF GREAT BRITAIN AND NORTHERN IRELAND

## Abstract

Female genital mutilation (FGM) remains prevalent in parts of Kenya, sustained by social norms around marriageability and social belonging. Although community interventions to end FGM are widely implemented, quantitative evidence of their effectiveness remains limited. This study evaluated whether mixed-sex community dialogues and women-only reflection circles implemented through the Girl Generation-Africa-Led Movement Programme changed FGM-related beliefs, intentions, social norms, and opposition to FGM in Isiolo County, Kenya. We analysed cross-sectional survey data from 2,235 respondents aged 15–60 years (1,314 women and 921 men). Exposure was self-reported participation in community dialogues and, among women, women-only reflection circles. Outcomes were pro-FGM beliefs, intention to cut a daughter, belief that uncut girls are not marriageable, and opposition to FGM. Associations were estimated using logistic regression with generalized estimating equations, adjusting for socio-demographic covariates. Among women, predicted probabilities were estimated across four exposure combinations, while a survey-weighted generalized structural equation model assessed pathways linking community dialogues to opposition to FGM. Participation in community dialogues was associated with lower odds of pro-FGM beliefs (aOR 0.49, 95% CI: 0.40-0.60), intentions to cut a daughter (aOR 0.34, 95% CI: 0.27-0.45), and belief that uncut girls are not marriageable (aOR 0.40, 95% CI: 0.31-0.53), and with higher odds of opposing FGM (aOR 3.09, 95% CI: 2.27-4.22). Among women, reflection circles primarily reduced intentions to cut and increasing opposition to FGM. Combined exposure produced the strongest effects, reducing the predicted probability of intending to cut a daughter from 38.6% to 12.7% and increasing opposition to FGM from 61.9% to 86.0%. Approximately 95% of the effect of community dialogues on opposition to FGM was mediated through changes in beliefs, intentions, and marriageability norms, with intentions representing the strongest pathway. These findings demonstrate that layered, community-led social norms interventions can meaningfully accelerate abandonment of FGM in high-prevalence settings.

## Introduction

Female genital mutilation (FGM) is a major public health and human rights concern. Globally, more than 230 million girls and women have undergone FGM with over half (144 million) happening in Africa, followed by Asia (over 80 million), and the middle East (over 6 million) [1]. Remarkably, every year, approximately 4 million girls globally are subjected to the practice [1]. FGM has no health benefits and is linked to severe immediate and long-term complications, including haemorrhage, infection, obstetric complications, sexual dysfunction, and psychological trauma [2]. In recognition of these harms, the global community envisages a world of zero new cases of FGM by 2030 under Sustainable Development Goal 5.3 [3].

Despite recent progress, FGM continues where it is deeply embedded in social norms. These norms shape ideas about marriageability, female sexuality, group identity, and moral virtue [4,5]. In many settings, families engage in FGM to avoid stigma, maintain social acceptance, or protect their daughters’ chances of marriage, even when they privately disagree with the practice [5]. Because of this, efforts to end FGM must engage with key stakeholders at the community level, rather than relying only on individual awareness or changes in the law.

Kenya has made important progress in reducing FGM. National prevalence has fallen from 38% in 1998 to 15% in 2022 [6]. However, there are still large within country disparities. Counties such as Isiolo, Mandera, Wajir, and Marsabit have higher FGM prevalence than the national average [6], driven by strong beliefs about purity, femininity, and marriageability [7]. In these settings, community-led behaviour change initiatives, such as dialogues, peer reflection groups, and participatory learning activities, are increasingly used to shift collective expectations and to strengthen people’s ability to resist the practice [5].

Evidence from social norms theory suggests that collective deliberation and public discussion can disrupt individual misperceptions about community support for harmful practices, build public consensus favouring abandonment, and generate coordinated behaviour change [8,9]. Interventions such as Tostan’s Community Empowerment Program [10] and other dialogue-based approaches have shown promise in improving knowledge, reshaping attitudes, and in some cases reducing the practice of FGM [11,12]. However, empirical data on how these interventions shift specific norm pathways, especially in a high-burden pastoralist contexts such as northern Kenya, remain limited.

From 2023 to 2024, The Girl Generation-Africa Led Movement (TGG-ALM) programme, supported by ActionAid International Kenya, implemented a layered package of mixed-sex community dialogues and women-only reflection circles in Isiolo County. These interventions aimed to challenge norms that support FGM, strengthen women’s agency, and build collective opposition to the practice. As part of the Population Council-led FGM Data Hub’s mandate of supporting the TGG-ALM programme in rigorous evidence generation, this study assesses the effectiveness of these interventions and how the layered package of interventions is linked to specific attitudes, intentions, and opposition to FGM in this setting.

The study uses data from a community-based survey of 2,235 men and women in Isiolo County to examine the effects of a layered community intervention strategy to shift FGM-related social norms. Community dialogues constituted the core intervention delivered to the general population, while women-only reflection circles were introduced as a targeted, additive component for women. The analysis addresses three questions. First, what is the association between participation in community dialogues and FGM-related norms and opposition to FGM in the general population? Second, among women, does participation in women-only reflection circles provide additional benefits beyond exposure to community dialogues alone, particularly for norms and intentions most closely tied to women’s agency? Third, through which pathways, do community dialogues influence opposition to FGM?

Answering these questions is important for at least two reasons. At the local level, the findings provide direct guidance for refining and scaling community-led norm-change strategies in Isiolo and similar high-prevalence counties. At the global level, the study adds empirical evidence on how layered social norms interventions operate, and on the mechanisms through which they may accelerate abandonment of FGM contributing to Sustainable Development Goal target 5.3 of ensuring zero new cases by 2030.

## Methods

### Ethics statement

The study was reviewed and approved by the Population Council’s Institutional Review Board (Protocol 1015), AMREF Ethics and Scientific Review Committee (AMREF-ESRC P1329/2022) and was granted administrative permission by the Kenya National Commission for Science, Technology, and Innovation (NACOSTI/P/24/35295). Before the interviews were conducted, verbal informed consent and assent were obtained from all participants. Participants were informed about the study and given the option to participate voluntarily. Those who agreed provided verbal informed consent, which was documented by the interviewer through an electronic confirmation indicating that the study had been explained and consent obtained. No written consent was collected to avoid creating identifiable records linking participants to the research. A waiver of documentation of consent (including assent for minors, where applicable) was granted by the relevant ethical review committee. This approach was justified on the grounds of protecting participant confidentiality and minimizing risk, given the sensitive and legally prohibited nature of female genital mutilation in the study context. Consistent with ethical guidance from the United Nations Department for Economic and Social Affairs on research involving violence against women and girls, verbal consent was deemed an appropriate and safer alternative, as written signatures could increase the risk of participant identification.

### Study setting

Isiolo county is located in Northern Kenya and is home to approximately 268,000 residents, with a predominantly pastoralist population spread across expansive arid and semi-arid lands. The county is ethnically diverse, comprising mainly Borana, Somali, and Samburu communities, each with longstanding cultural traditions and social structures that shape gender norms, rites of passage, and expectations around marriage [13]. Livelihoods are centred on pastoralism, and small-scale commerce, with households often experiencing recurrent climate shocks (extended droughts and flash floods) leading to limited access to services and disruption to livelihoods [13].

FGM remains deeply embedded in cultural and social life in parts of Isiolo. Historical data and programme reports indicate that many communities still regard FGM as a prerequisite for marriage, safeguarding purity and guaranteeing social acceptance [13]. Kenya’s national FGM prevalence has fallen to 15%, but progress in Isiolo has been slower and prevalence is estimated at 66% [6]. This persistence reflects strong kinship networks, intergenerational transmission of norms and the limited reach of structural interventions such as formal education and gender-transformative programming in this context. These dynamics make Isiolo county a critical setting for examining community-led norm-change interventions.

### Community interventions in Isiolo

The Girl Generation-Support to the Africa-Led Movement (TGG-ALM) programme implemented community-based social norms interventions in Isiolo aimed at accelerating abandonment of FGM [5]. Interventions were implemented by ActionAid International–Kenya and consisted of two core components: community intergenerational dialogues and women-only reflection circles. Community dialogues were open, mixed-sex forums that brought together women, men, youth, elders, and other influential community members [5]. These dialogues were designed to facilitate collective reflection on FGM, gender norms, marriageability expectations, and the social consequences of cutting. Sessions emphasized participatory discussion, storytelling, and deliberation, enabling participants to surface shared beliefs, question perceived social expectations, and discuss pathways toward collective abandonment of FGM.

Women-only reflection circles were introduced later as a complementary, gender-specific component layered on top of the community dialogues. These circles provided women and adolescent girls with a safe, single-sex space to reflect more deeply on FGM, bodily autonomy, motherhood, and decision-making around daughters [5]. The intent was not to replace community dialogues, but to strengthen norm change among women by addressing constraints related to voice, power, and social risk that may limit women’s participation or expression in mixed-gender settings [5]. Participation in women-only circles therefore occurred in a context where broader community dialogues were already ongoing.

Participants were recruited through community-based mobilization led by local facilitators in collaboration with village leaders and existing administrative structures. Open invitations and targeted outreach were used to ensure balanced participation across women, men, adolescents, and influential community members. Additional efforts focused on including underrepresented groups, particularly young people and marginalized women. Women-only reflection circles specifically engaged women and adolescent girls, including those less likely to speak in mixed-gender settings, ensuring more equitable participation across the interventions.

### Study population and sampling

This study uses cross-sectional survey data collected between June and July 2024. The study population comprised 2,235 respondents aged 15–60 years of whom 1,314 were women and 921 were men.

We employed a multistage cluster sampling design in which wards and villages were first randomly selected as primary sampling units, followed by systematic selection of households within each cluster. Within each selected household, one eligible respondent was randomly chosen using a household roster. Because the sampling strategy applied a fixed number of households per cluster and random selection of one respondent per household, the probability of selection at each stage was approximately equal across individuals. As a result, the survey was treated as self-weighting, and analyses were conducted using equal weights.

While variation in cluster sizes can, in principle, introduce unequal selection probabilities in multistage designs, the use of fixed sampling fractions within clusters and comparable cluster sizes in the study setting minimised this risk.

### Data collection

Data were collected electronically using Open Data Kit (ODK) on tablets. Interviews were conducted in English, Kiswahili and local languages (Somali, Borana and Samburu). Enumerators received training on research ethics, interviewing on sensitive topics and maintaining confidentiality. The questionnaire predominantly used vignette-based indirect questions to reduce social desirability bias. Survey modules covered demographic characteristics, attitudes towards cut versus uncut girls, perceptions of social norms within families, peer groups and the wider community, intentions regarding cutting daughters and perceived marriageability, opposition to FGM, and self-reported exposure to intervention activities.

### Measures

#### Exposure variables.

*Community dialogues (CD)-* Defined as a binary indicator of attendance/participation in mixed-community dialogue sessions. Coded 1 if a respondent attended at least one session and 0 otherwise.

*Women’s reflection circles-* Defined as a binary indicator of attendance for women only. Coded 1 if a woman respondent attended at least one session of women’s reflection circles and 0 otherwise.

#### Outcome variables.

The analysis focused on four binary outcomes, coded as described in S1 Table. First, we measured beliefs favourable to cutting using an attitudinal index based on five items capturing perceived associations between FGM and health, cleanliness, purity, modernity, and women’s social standing. Each item was dichotomised such that a value of 1 indicated endorsement of a belief supportive of FGM. Dichotomisation was applied to reduce measurement noise arising from highly skewed response distributions, as endorsement of at least one pro-FGM belief was nearly universal in the sample (97.05%), limiting the discriminatory power of individual items.

The five items were summed to generate a composite score ranging from 0 to 5. The scale demonstrated good internal consistency (Cronbach’s α = 0.74), with item-test correlations ranging from 0.63 to 0.78 and item-rest correlations from 0.40 to 0.61, indicating that all items contributed meaningfully to a common underlying construct (S2 Table). No item deletion improved scale reliability.

To enhance interpretability and distinguish respondents with a predominantly pro-FGM normative orientation from those expressing isolated or ambivalent agreement, the index was dichotomised at a threshold of three or more endorsed items. This threshold reflects majority endorsement of pro-FGM beliefs and is consistent with the observed distribution of the scale, which was centred around values of 3–4.

The second outcome was intention to cut a daughter defined as 1 if a respondent indicated any likelihood of planning to cut daughter. The third was endorsement of the marriageability norm, defined as agreement with the statement that “uncut girls are not marriageable.” The fourth outcome captured opposition to FGM and defined as 1 if a respondent indicated that they were against the practice and 0 otherwise.

#### Covariates.

The analysis included several pre-specified sociodemographic covariates to account for characteristics that may influence FGM-related attitudes, perceived norms and behaviours. These covariates were selected based on theoretical relevance and evidence from prior research on gender norms and harmful practices. These included sex, marital status and education level, to capture differences in social roles, decision-making power and exposure to information. Age was grouped into four categories (15–19, 20–24, 25–35 and 36–60 years) to reflect distinct life-course stages in which attitudes and experiences related to FGM may differ. Ethnicity (defined categorically and includes Somali, Borana and Samburu) and religion (Christian/other or Muslim) were incorporated to control for cultural and normative contexts, given their strong influence on the practice and its social meaning in the study settings.

### Statistical analysis

All analyses accounted for the stratified, multistage sampling design and clustering at the village level. Owing to all eligible individuals having the same probability of selection, the survey was treated as self-weighting, and analyses were conducted using equal weights across respondents. The analysis was conducted in six steps:

First, we began by describing the sample using proportions with 95% confidence intervals for sociodemographic characteristics and each outcome. Secondly, we examined the extent to which the four binary outcomes captured related underlying constructs by estimating tetrachoric correlations and presenting the resulting 4 × 4 correlation matrix. Third, associations between exposure to community dialogues and each outcome were estimated using logistic regression models with village-level clustering and robust standard errors, implemented through generalized estimating equations. Fourth, limiting the sample to women only, we tested the additive impact of women reflection circles on the four outcomes.

**Fig 1:**
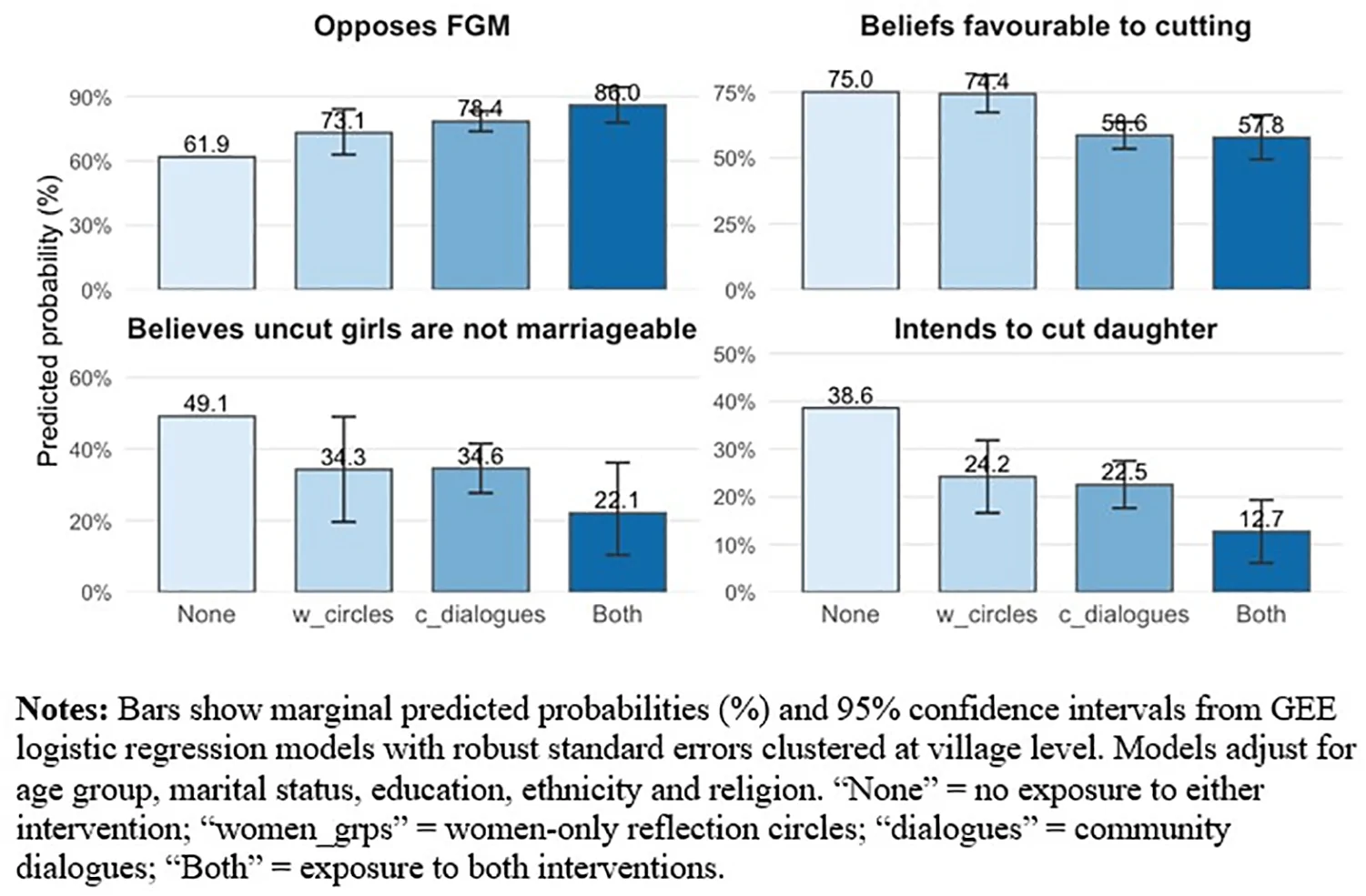
Additive effects of community dialogues and women’s reflection circles on FGM norms and opposition.

Fifth, because four correlated outcomes were analysed for each exposure, we corrected for multiple testing using both the Westfall-Young resampling procedure (100 permutations) and the Bonferroni method. Sixth, from the women only sample models, we generated predicted probabilities for four exposure categories, no intervention, women-only circles, community dialogues only, and both interventions combined, and report percentage-point differences with 95% confidence intervals (Fig 1; S3 Table).

Lastly, to examine how the association between community dialogues and opposition to FGM is distributed across related intermediate variables, we estimated a generalized structural equation model (GSEM) with Bernoulli-logit links. The model included beliefs favourable to cutting, intention to cut a daughter, and perceived marriageability as intermediate variables based on a priori theoretical frameworks. Given the cross-sectional design, these estimates do not imply causal mediation but rather describe how associations between exposure and outcome are statistically distributed across correlated constructs. All mediation results should therefore be interpreted as theory-informed associative decompositions rather than evidence of causal mechanisms. All inference used two-sided tests with α = 0.05 and 95% confidence intervals adjusted for clustering.

Analyses were conducted using complete-case estimation, and effective sample sizes therefore vary across models depending on item response. As some modules and outcomes were not completed by all participants, the sample sizes for individual models differed and were reported alongside the respective results.

## Results

### Sample descriptive statistics

The analytic sample comprised 2,235 respondents, of whom 1,314 (58.8%) were women and 921 (41.2%) were men (Table 1). Age was broadly distributed, with 11.9% aged 15–19 years, 15.7% aged 20–24 years, 39.4% aged 25–35 years, and 36–60 years constituting the remainder. Most respondents were married (64.7%), while 35.3% were never married or separated. Lack of education among respondents was substantially high with 32.3% having no formal education, 30.6% had attained primary, 28.6% secondary, and 8.5% tertiary education. By ethnicity, 7.6% identified as Somali, 61.7% as Borana and 30.7% as Samburu/other groups. By religion, 79.1% were Muslim or other religion and 20.9% were Christian.

**Table 1:** Key sample characteristics.

Variable	Weighted %	95% CI	N
**Socio-demographic characteristics**			
Sex			
Male	41.2	(40.5-41.9)	921
Female	58.8	(58.1-59.5)	1,314
Age			
15-19	11.9	(10.1-14.1)	263
20-24	15.7	(14.2-17.3)	345
25-35	39.4	(37.2- 41.6)	867
36-60	33.0	(30.8-35.2)	726
Marital Status			
Separated/Never married	35.3	(32.3-38.5)	778
Married	64.7	(61.5-67.7)	1,423
Education level			
No education	32.3	(28.2- 36.8)	711
Primary education	30.6	(28.1-33.3)	674
Secondary education	28.6	(24.7-32.8)	629
Tertiary	8.5	(7.1-10.1)	187
Ethnicity			
Somali	7.6	(6.6 – 8.8)	170
Borana	61.7	(59.7 – 63.7)	1,380
Samburu/ Other	30.7	(28.8 – 32.6)	685
Religion			
Muslim	70.4	(64.5- 75.7)	1,549
Christian/Other	29.6	(24.3- 35.5)	651
**Outcomes**			
Beliefs favourable to cutting			
None to low (0–2)	36.4	(32.9- 40.0)	813
Mixed to extreme (3–5)	63.6	(60.0-67.1)	1,322
Intends to cut daughter	32.0	(28.5-35.6)	886
Believes that uncut girls are not marriageable	31.1	(36.3-42.0)	915
Oppose FGM	64.1	(59.7-68.4)	1,410
**Community norm shifting intervention attendance**			
Attended community dialogues	18.7	(15.2-22.9)	419
Attended women only dialogues	9.9	(7.5-13.0)	130
Attended both community and women only dialogues (limited to women only)	6.2	(4.1-9.1)	81

Attitudes and intentions related to FGM showed substantial heterogeneity. Almost two-thirds (63.6%) of the respondents expressed mixed or positive attitudes towards cut girls. About one-third (31.1%) believed that uncut girls are not marriageable, whereas 64.1% opposed FGM. Exposure to community norm-shifting activities was modest: 18.7% attended mixed-community dialogues, 9.9% attended women-only dialogues, and 6.2% attended both (among women).

### Correlations among binary outcomes

The tetrachoric correlations indicate strong co-movement among pro-FGM attitudes and intentions outcomes (Table 2). Beliefs favourable to cutting correlate positively with intentions to cut a daughter (ρ = 0.55), and the belief that uncut girls are not marriageable is even more strongly aligned with those intentions (ρ = 0.69). Opposition to FGM, shows the opposite pattern, with a very strong negative link to intentions (ρ = -0.88) and moderate negative links to both pro-FGM beliefs (ρ = -0.48 and -0.60).

**Table 2:** Tetrachoric correlation matrix among outcomes.

Outcome	Beliefs favourable to cutting	Intends to cut daughter	Agrees that uncut girls are not marriageable	Opposes FGM
Beliefs favourable to cutting	1			
Intents to cut daughter	0.55	1.00		
Believes that uncut girls are not marriageable	0.30	0.69	1.00	
Oppose FGM	-0.48	-0.88	-0.60	1.00

**Notes:** Entries are tetrachoric correlation coefficients estimated between binary outcomes. Positive correlations therefore indicate co-occurrence of similar tendencies (for example, pro-FGM beliefs and intention to cut), while negative correlations indicate that endorsing a pro-FGM norm is associated with lower opposition to FGM. All correlations are statistically significant at p < 0.001.

### Associations between community dialogues and FGM norms

Participation in community dialogues was strongly associated with more progressive norms across all four outcomes (Table 3 and S1 Fig). After adjusting for sex, age, marital status, education, ethnicity, and religion and clustering by village, dialogues were associated with substantially lower odds of endorsing pro-FGM beliefs (aOR = 0.49; 95% CI: 0.40-0.60; p < 0.001), lower intentions to cut a daughter (aOR = 0.34; 95% CI: 0.27-0.45; p < 0.001), and lower agreement that uncut girls are not marriageable (aOR = 0.40; 95% CI: 0.31-0.53; p < 0.001). Dialogues were also linked to markedly higher likelihood to oppose FGM (aOR = 3.09; 95% CI: 2.27-4.22; p < 0.001). These associations were robust to family-wise error correction: for all four outcomes, adjusted p-values for community dialogues remained <0.001 across Westfall-Young and Bonferroni procedures (S3 Table).

**Table 3:** Generalised estimation equation models for the association of community dialogues with FGM norms and opposition.

Variables	Beliefs favourable to cutting		Intends to cut daughter		Agrees that uncut girls are not marriageable		Opposes FGM	
	AOR	P-value	AOR	P-value	AOR	P-value	AOR	P-value
	(95% CI)		(95% CI)		(95% CI)		(95% CI)	
Community dialogues	0.49 (0.40–0.60)	<0.001	0.34 (0.27–0.45)	<0.001	0.40 (0.31–0.53)	<0.001	3.09 (2.27–4.22)	<0.001
Gender (Ref: male)								
Women	2.44 (2.01–2.97)	<0.001	1.28 (0.88–1.84)	0.192	1.69 (1.15–2.46)	0.007	1.00 (0.65–1.53)	0.988
Age (Ref:15–19)								
20-24	0.97 (0.73–1.30)	0.855	1.65 (1.00–2.66)	0.052	0.85 (0.54–1.34)	0.476	0.63 (0.42–0.95)	0.029
25-35	1.36 (0.93–1.99)	0.128	1.61 (1.09–2.41)	0.017	0.74 (0.44–1.25)	0.257	0.64 (0.43–0.96)	0.029
36-60	1.78 (1.18–2.68)	0.006	1.83 (1.22–2.75)	0.004	0.77 (0.46–1.31)	0.338	0.41 (0.26–0.63)	<0.001
Marital Status (Ref: Separated/Never married)								
Married	0.91 (0.70–1.17)	0.449	0.81 (0.65–1.02)	0.072	0.95 (0.74–1.21)	0.675	1.03 (0.80–1.33)	0.804
Education level (Ref: no education)								
Primary education	0.89 (0.66–1.20)	0.442	0.62 (0.46–0.87)	0.005	0.63 (0.47–0.84)	0.002	1.68 (1.24–2.27)	0.001
Secondary education	1.05 (0.72–1.55)	0.787	0.32 (0.22–0.45)	<0.001	0.45 (0.31–0.66)	<0.001	2.58 (1.87–3.56)	<0.001
Tertiary	1.25 (0.79–1.97)	0.344	0.31 (0.19–0.50)	<0.001	0.28 (0.14–0.54)	<0.001	3.47 (2.20–5.47)	<0.001
Ethnicity (Ref: Somali)								
Borana	1.05 (0.63–1.75)	0.847	0.57 (0.36–0.92)	0.020	0.83 (0.51–1.34)	0.444	1.78 (1.04–3.03)	0.035
Samburu/Other	2.60 (1.30–5.24)	0.008	0.74 (0.40–1.37)	0.340	3.15 (1.45–6.85)	0.004	3.34 (1.56–7.15)	0.002
Religion (Ref: Christian)								
Muslim/other religion	1.85 (1.04–3.27)	0.036	2.09 (1.52–2.88)	<0.001	2.00 (1.44–2.79)	<0.001	0.70 (0.53–0.93)	0.014
**Number of observations**	**2,200**		**2,197**		**1989**		**2,197**	

**Notes:** Adjusted odds ratios (AORs) are from population-averaged Generalised Estimating Equation (GEE) logistic regression models with robust standard errors clustered at the village level. All models use an exchangeable correlation structure and adjust for gender, age group, marital status, education, ethnicity, and religion. P-values correspond to two-sided Wald tests. Confidence intervals are shown in parentheses. The number of observations varies across models due to item-level non-response.

Women had higher odds than men of holding pro-FGM beliefs (aOR = 2.44; 95% CI: 2.01-2.97; p < 0.001) and of believing that uncut girls are not marriageable (aOR = 1.69; 95% CI: 1.15-2.46; p = 0.007) but they did not differ significantly from men in intentions to cut or opposition to FGM (both p > 0.19). Age patterns were more pronounced for older adults. Participants aged 25–35 and 36–60 had higher odds of intending to cut a daughter compared with those aged 15–19 (aOR = 1.61; 95% CI: 1.09-2.41; p = 0.017 and aOR = 1.83; 95% CI: 1.22-2.75; p = 0.004, respectively). The oldest group (36–60) also had higher odds of pro-FGM beliefs (aOR = 1.78; 95% CI: 1.18-2.68; p = 0.006). Older age groups showed reduced opposition to FGM relative to adolescents, with the strongest reduction among those aged 36–60 (aOR = 0.41; 95% CI: 0.26-0.63; p < 0.001). Education was protective against harmful norms, except in the case of pro-FGM beliefs. Compared with respondents with no schooling, those with primary, secondary, and tertiary education had substantially lower odds of intending to cut a daughter (aOR = 0.62; 95% CI: 0.46-0.87; p = 0.005; 0.32; 95% CI: 0.22-0.45; p < 0.001; and 0.31; 95% CI: 0.19-0.50; p < 0.001, respectively) and of agreeing that uncut girls are not marriageable (aOR = 0.63; 95% CI: 0.47-0.84; p = 0.002; 0.45; 95% CI: 0.31-0.66; p < 0.001; and 0.28; 95% CI: 0.14-0.54; p < 0.001). Education was also consistently associated with greater opposition to FGM with progressively higher odds at each higher schooling level achieved (aOR = 1.68 for primary; 2.58 for secondary; and 3.47 for tertiary; all p ≤ 0.001).

Ethnic and religious differences were also evident. Compared with Somali respondents, Borana participants had lower odds of intending to cut (aOR = 0.57; 95% CI: 0.36-0.92; p = 0.020) and higher opposition to FGM (aOR = 1.78; 95% CI: 1.04-3.03; p = 0.035), while those from Samburu/other groups had higher odds of pro-FGM beliefs (aOR = 2.60; 95% CI: 1.30-5.24; p = 0.008), greater endorsement of marriageability norms (aOR = 3.15; 1.45-6.85; p = 0.004), and higher opposition to FGM (aOR = 3.34; 95% CI: 1.56-7.15; p = 0.002). Relative to Christians, respondents who were Muslim or from other religions had higher odds of pro-FGM beliefs, intentions to cut, and marriageability beliefs (aORs 1.85-2.09; all p ≤ 0.036), and lower odds of opposing FGM (aOR = 0.70; 95% CI: 0.53-0.93; p = 0.014).

### Additive associations of community dialogues and women’s reflection circles on FGM norms and opposition to FGM

Fig 1 (full estimates in S4 Table) presents the adjusted predicted probabilities associated with exposure to community dialogues and women’s reflection circles, delivered alone and in combination, in relation to women’s beliefs, intentions, and opposition to FGM. Estimates are expressed as predicted probabilities with absolute percentage-point (pp) differences relative to no reported exposure, alongside 95% confidence intervals and p-values. Multiple-testing adjustments using Westfall-Young and Bonferroni procedures, reported in S3 Table, confirmed the robustness of associations for community dialogues across all four outcomes, but indicated a more selective pattern for women’s reflection circles, with only intention to cut remaining statistically significant after correction.

First, beliefs favourable to cutting were still very common among women with no exposure to either intervention (75.0%). Participation in women’s reflection circles alone did not meaningfully change this (74.4%; -0.6 percentage points [pp], 95% CI: 7.7 to 6.5; p = 0.869). In contrast, community dialogues were associated with a large and statistically significant reduction (58.6%; -16.4 pp, -21.5 to -11.3; p < 0.001). The combined package produced the largest decline (57.8%; -17.1 pp, -25.5 to -8.7; p < 0.001). Second, intention to cut a daughter was considerably more responsive. From a baseline probability of 38.6% with no intervention, women’s reflection circles alone reduced intent to 24.2% (-14.4 pp, -22.0 to -6.8; p < 0.001), and community dialogues alone reduced it to 22.5% (-16.0 pp, -21.0 to -11.1; p < 0.001). Third, the belief that “uncut girls are not marriageable” followed a similar graded pattern. Relative to 49.1% among women with no intervention, women’s reflection circles reduced this belief to 34.3% (-14.8 pp, -29.5 to -0.23; p = 0.046) and community dialogues to 34.6% (-14.5 pp, -21.4 to -7.6; p < 0.001). When both platforms were combined, the probability fell to 22.1% (-27.0 pp, -39.7 to -14.1; p < 0.001). Finally, women’s opposition to FGM also increased in a dose-response fashion. From 61.9% among those with no exposure, opposition to FGM rose to 73.1% with women’s reflection circles (+11.2 pp, 1.0 to 21.0; p = 0.032) and to 78.4% with community dialogues (+16.5 pp, 12.2 to 20.8; p < 0.001). The combined intervention produced the largest gain, with opposition reaching 86.0% (+24.1 pp, 15.9 to 32.4; p < 0.001).

### Component analysis of the association of community dialogue on opposition towards FGM

Table 4 presents mediation results examining how the association between community dialogues (CD) and opposition to FGM operates through three intermediate variables: beliefs favourable to cutting, intention to cut a daughter, and the marriageability norm. The diagram in S1 Fig summarises the corresponding logit coefficients from this model.

**Table 4:** Mediation of community dialogues on respondent’s opposition to FGM (survey-weighted GSEM, log-odds).

	Effect	Coef (log-odds)	p-value	95% CI
**Main effects**	NDE: Direct effect (Dialogue → Oppose FGM)	0.47	0.031	(0.04 to 0.90)
	NIE: Total indirect effect (via mediators)	8.24	<0.001	(6.15 to 10.34)
	TE: Total effect (NDE + NIE)	8.72	<0.001	(6.55 to 10.88)
**Path-specific indirect effects**	Path 1: Dialogue → Beliefs → Intent → Opposition	3.76	<0.001	(2.25 to 5.27)
	Path 2: Dialogue → Beliefs → Uncut not marriageable → Opposition	0.52	<0.001	(0.28 to 0.76)
	Path 3: Dialogue → Intent → Opposition	2.55	<0.001	(1.78 to 3.33)
	Path 4: Dialogue → Uncut not marriageable → Opposition	0.89	<0.001	(0.51 to 1.26)
	Path 5: Dialogue → Beliefs → Opposition	0.53	<0.001	(0.26 to 0.79)
**Shares of total indirect effect**	Share mediated by Path 1 (Beliefs → Intent → Opposition)	0.46	<0.001	(0.36 to 0.55)
	Share mediated by Path 2 (Beliefs → Uncut not marriageable → Opposition)	0.06	<0.001	(0.04 to 0.09)
	Share mediated by Path 3 (Intent → Opposition)	0.31	<0.001	(0.24 to 0.38)
	Share mediated by Path 4 (Uncut not marriageable → Opposition)	0.11	<0.001	(0.05 to 0.16)
	Share mediated by Path 5 (Beliefs → Opposition)	0.06	<0.001	(0.04 to 0.09)
**Overall proportion mediated (of total effect)**	Indirect/ (Indirect + Direct)	0.95	<0.001	(0.90 to 0.99)

**Notes:** Estimates are from a survey-weighted generalized structural equation model and are reported on the log-odds scale. NDE denotes the direct effect of community dialogues on opposition to FGM, NIE the indirect effect operating through changes in beliefs, intentions to cut, and perceptions of marriageability, and TE the sum of direct and indirect effects. Path-specific effects decompose the indirect effect into distinct mediation pathways, and the share mediated indicates each pathway’s contribution to the total indirect effect. Because the model is nonlinear, coefficients are best interpreted in terms of direction and relative magnitude rather than exponentiated odds ratios.

The mediation results indicate that community dialogues were significantly associated with higher opposition to FGM through both direct and indirect components within the model. The estimated direct association between community dialogues and opposition to FGM was statistically significant (NDE = 0.47 log-odds; OR= 1.60; p = 0.031). The combined indirect associations operating through beliefs, intentions, and the marriageability norm were substantially larger (NIE = 8.24; p < 0.001), with an overall estimated association of 8.72 log-odds (TE = 8.72; p < 0.001). Approximately 95% of the total estimated association between community dialogues and opposition to FGM was captured by the specified intermediate variables in the model.

Decomposition of the indirect component suggests that the strongest associations were observed along sequences linking beliefs and intentions. The largest contribution was observed for the sequence linking community dialogues, beliefs, intentions, and opposition (β = 3.76; p < 0.001), followed by the sequence linking community dialogues, intentions, and opposition (β = 2.55; p < 0.001). Sequences involving the marriageability norm also showed statistically significant associations, including those linking community dialogues to the perception that uncut girls are not marriageable and subsequently to opposition (β = 0.89; p < 0.001), as well as those operating through beliefs and marriageability perceptions (β = 0.52; p < 0.001). A smaller but statistically significant association was observed for the link between beliefs and opposition (β = 0.53; p < 0.001).

In terms of relative contributions within the model, approximately 46% of the total indirect association was accounted for by the sequence linking beliefs, intentions, and opposition, while 31% was associated with the link between intentions and opposition. Associations involving the marriageability norm accounted for approximately 17% of the total indirect component (11% through the marriageability-opposition link and 6% through beliefs and marriageability), with the remaining 6% corresponding to the association between beliefs and opposition.

## Discussion

This study provides new empirical evidence on how layered community-based interventions can shift FGM-related norms, intentions, and opposition to FGM in a high-prevalence pastoralist setting. Consistent with social norms theory [9], participation in mixed-sex community dialogues was strongly associated with less favourable beliefs about cutting, reduced intention to cut a daughter, lower endorsement of the marriageability norm, and substantially higher opposition against the practice. Women-only reflection circles, while more selective in associations, contributed additional gains in intentions and increasing opposition to FGM, and in combination with community dialogues, produced the strongest improvements across nearly all outcomes. Importantly, the mediation analysis highlights that the association of community dialogues on respondent’s opposition to FGM operates predominantly through shifts in beliefs and intentions.

These findings align with a robust body of literature showing that FGM persists where it is embedded in interdependent expectations about marriageability, purity, and social belonging [4,5,14,15]. In Isiolo, as in many practising communities, parents may support abandonment privately yet still feel compelled to conform to perceived community expectations [13]. Social norms-oriented interventions therefore aim not only to change individual attitudes but to transform the collective expectations that sustain the practice [5]. For example, evidence from dialogue-based programmes such as Tostan’s Community Empowerment Program demonstrates that when communities are given structured opportunities for collective reflection, misperceptions about others’ support for FGM decline and coordinated abandonment becomes possible [10]. The strong associations observed in this study, particularly for the marriageability norm and intentions, reinforce these insights and illustrate the relevance of such approaches in pastoralist contexts where kinship influence, social surveillance, and gendered expectations remain strong. These findings align with the theories around stages of change and its application to FGM [16,17].

Another important finding relates to gender differences in normative beliefs and expressed positions on FGM. Women in this study had higher odds of endorsing pro-FGM beliefs than men yet did not differ significantly in intentions to cut a daughter or in opposition to the practice. This apparent divergence highlights the distinction between internalised norms, behavioural intentions, and expressed positions in contexts where social expectations are strongly enforced. In high-prevalence settings, FGM is widely understood as a social norm sustained by expectations around marriageability, social acceptance, and group belonging, where families conform to what they believe others expect of them [18]. Women, as primary custodians of cultural practices, may therefore internalise these norms more strongly through processes of socialisation and intergenerational transmission, even where private attitudes are shifting [19].

At the same time, decision-making around FGM is rarely individual and is typically embedded within broader household and kinship structures involving elders, male relatives, and extended social networks. In such contexts, women’s ability to translate beliefs into intentions or openly expressed opposition may be constrained by limited agency and the risk of social sanctions. Qualitative and social norms research has shown that individuals, particularly women, may align their expressed attitudes with perceived community expectations, even when they privately disagree, due to the high social costs of non-conformity [20]. This helps explain why higher endorsement of pro-FGM beliefs among women does not necessarily translate into differences in behavioural intentions or opposition.

Generational dynamics may further contribute to this pattern as the results show. Older cohorts, who were socialised in contexts where FGM was more strongly institutionalised, may retain more entrenched beliefs even as broader community attitudes begin to shift [4]. However, shifts in intentions and expressed opposition may occur more uniformly across men and women where decision-making remains collective and contingent on wider social consensus.

More broadly, this pattern reflects a well-documented feature of norm-driven practices: changes in personal beliefs do not automatically translate into behavioural change where collective expectations remain intact. Evidence from multiple settings indicates that while many individuals’ express opposition to FGM, behaviour often lags due to persistent social pressures and coordination constraints [21].

The findings also offer insight into other persistent demographic differences. Education demonstrated a clear protective gradient; even modest schooling was associated with lower harmful intentions and higher willingness to act against FGM. This mirrors evidence from Kenya and elsewhere that education broadens social networks, increases exposure to alternative norms, and strengthens autonomy in decision-making [22,23]. Ethnic and religious differences also highlight the need for culturally grounded programming that attends to diverse interpretations of FGM’s moral and social significance within multi-ethnic counties such as Isiolo [12].

This study makes three key contributions to the FGM norms literature. First, by examining multiple outcomes simultaneously, it demonstrates how attitudes, intentions and perceived norms move together. The strong tetrachoric correlations reflect the normative architecture described in previous work, in which beliefs about the benefits of cutting, expectations of others’ behaviour and intentions to conform form a coherent system [9,24,25]. Willingness to act against FGM stands in sharp contrast to these elements, consistent with research showing that willingness to act requires both revised personal attitudes and a belief that community support for change exists [26].

Second, this analysis distinguishes the effects of two complementary interventions. While most evaluations treat community dialogues as a single unit, findings from this study show that mixed-sex dialogues appear to shift broader normative expectations, whereas women-only reflection circles reinforce individual self-efficacy and decision-making. This aligns with calls for layered norm-change strategies that engage both women and wider social networks, including men, elders and community leaders, whose influence is central in settings with strong kinship and pastoralist governance structures [4,27].

Third, the mediation analysis clarifies how changes in opposing FGM emerge. Beliefs and intentions served as dominant pathways linking community dialogues to opposition to FGM. This echoes theoretical models outlining how personal normative beliefs, perceived community norms and anticipated sanctions jointly shape readiness to intervene [24,28]. No prior study in Kenya has quantified such pathways using structural modelling, making this an important methodological and conceptual contribution.

### Strengths and limitations

This study contributes to the evidence base on social norm change by examining not only whether community dialogues are associated with opposition to FGM, but also how these associations operate through specific normative pathways. By combining mediation analysis with population-averaged generalized estimating equations, the analysis moves beyond single-equation associations to test theory-driven mechanisms linking dialogue exposure to beliefs, intentions, and opposition to FGM. The consistency of findings across multiple related outcomes and pathways strengthens confidence in the internal coherence of the results.

Several limitations should nevertheless be considered. Although vignette questions were included to reduce social desirability bias, some residual bias may remain, as is common in surveys on sensitive practices such as FGM [9]. In addition, the analysis draws on data from a single pastoralist county, and findings may not fully generalise to other Kenyan contexts where social organisation and programme implementation differ.

The cross-sectional design limits causal inference. However, exposure to community dialogues preceded the measurement of beliefs, intentions, and opposition outcomes within the programme design, providing a plausible temporal ordering of constructs despite their observation at a single time point. Moreover, the use of theoretically specified mediation pathways and the coherence of results across multiple outcomes reduce the likelihood that findings reflect spurious associations alone. Longitudinal data would nonetheless be required to confirm causal direction, assess norm diffusion beyond direct participants, and examine the durability of change over time.

Despite these limitations, the findings reinforce the importance of community-led norm change as a central strategy for accelerating progress towards Kenya’s goal of eliminating FGM by 2030 [22]. Future research should prioritise longitudinal or panel designs to track normative dynamics, explore diffusion processes within communities, and assess the sustainability of change after programme cycles end, an evidence gap highlighted in global reviews [27]. Further work is also needed to understand how men’s participation, intergenerational dialogue, and community leadership can be more effectively leveraged, given their central role in shaping and enforcing social expectations [4,26].

## Conclusion

This study shows that layered community interventions in form of community dialogues and women’s reflection circles can meaningfully shift attitudes, intentions and opposition to FGM. By illuminating the pathways through which these interventions influence social norms and behaviours, it contributes to the growing body of evidence that coordinated, gender-transformative, community-led interventions are essential for reducing the practice. These findings reinforce theoretical and empirical work on social norms and provide actionable insights for practitioners and policymakers seeking to accelerate the abandonment of FGM in high-prevalence settings.

## Supporting information

S1 Table Outcomes variable definition. (DOCX)

S2 Table Family-wise adjusted associations of community dialogues and women’s reflection circles with outcomes. (DOCX)

S3 Table Additive effects of women’s reflection circles and community dialogues on FGM norms and opposition among women. (DOCX)

S4 Table Internal consistency and reliability of FGM beliefs scale. (DOCX)

S1 Fig Pathways from community dialogues to opposing FGM: Survey GSEM results. (DOCX)
