# Supplementary material for: Shifting social norms, behavioural intentions and opposition to female genital mutilation: Effectiveness of layered community interventions in a high prevalence setting in Kenya
Source: PLOS Glob Public Health. 2026 Sep 10;6(9):e0007044. doi: 10.1371/journal.pgph.0007044 (PMC13561337; doi:10.1371/journal.pgph.0007044)
Supplement: Supplementary file 1 [file pgph.0007044.s001.docx]

**S1 Table. Outcomes variable definition**

| **Variable** | **Definition and coding** |
| --- | --- |
| **Beliefs about uncut girls** | **Violence-endorsing attitudes**   1. A girl who has undergone circumcision is healthy 2. A girl who has undergone circumcision is clean 3. A girl who has undergone circumcision is pure 4. A girl who has undergone circumcision is equal to other females 5. A girl who has undergone circumcision is modern   Each of the questions except question number 4 which was reverse coded, were coded 1 if the respondent responded (neutral/agree/strongly agree) and 0 if the respondent indicated (disagree/strongly disagree)  An index was then created from the five indicators ranging between 0-5 with 0 indicating zero positive perceptions on cut girls and 5 extreme positive perception. Binary measure reporting at least 3 of the 5 items was created. Variable was coded as 1 if participants reported at least three of the five items, and 0 if they reported two or less of the items. |
| **Intention to cut** | Imagine you have a daughter who has reached the age at which a girl custom, how likely are you to cut the daughter?  Binary variable coded 1 if respondent indicated (neutral/likely/ very likely), 0 if respondent said (unlikely/very unlikely) |
| **Believes that men are unlikely to marry uncut women** | What is the likelihood that men in your community are willing to marry uncut women?  Binary variable coded 1 if respondent indicated (neutral/unlikely/ very unlikely), 0 if respondent said (likely/very likely) |
| **Opposition to FGM** | How would you rate your opposition to or support of FGM?  Binary variable coded 1 if respondent indicated completely oppose FGM and 0 otherwise |
