# Supplementary material for: Shifting social norms, behavioural intentions and opposition to female genital mutilation: Effectiveness of layered community interventions in a high prevalence setting in Kenya
Source: PLOS Glob Public Health. 2026 Sep 10;6(9):e0007044. doi: 10.1371/journal.pgph.0007044 (PMC13561337; doi:10.1371/journal.pgph.0007044)
Supplement: Supplementary file 2 [file pgph.0007044.s002.docx]

**Multiple outcome testing**

S2 Table summarises family-wise error–corrected p-values for all GEE models in the full sample and for women-only models. Across both specifications, community dialogues are consistently and strongly associated with more progressive FGM norms and higher opposition. For every outcome, the p-values for community dialogues remain <0.001 under the conventional, Westfall-Young and Bonferroni adjustments, providing robust evidence that participation in dialogues is linked to lower pro-cutting beliefs, reduced intention to cut a daughter, weaker endorsement of the view that uncut girls are not marriageable, and greater opposition to FGM. Women’s reflection circles show a more selective pattern. In the women-only models, participation in reflection circles is strongly associated with lower intention to cut a daughter, with all p-values ≤0.01 even after multiple-testing correction. By contrast, there is no indication that women’s reflection circles affect general pro-cutting beliefs. For marriageability beliefs and opposition to FGM, the conventional and Westfall-Young p-values suggest possible benefits, but these associations do not remain statistically significant once the stricter Bonferroni adjustment is applied, indicating more modest and less certain effects than for community dialogues

**S2 Table. Family-wise adjusted associations of community dialogues and women’s reflection circles with outcomes**

| **Outcomes** | **All sample model** | | | **Women only model** | | | | | | |
| --- | --- | --- | --- | --- | --- | --- | --- | --- | --- | --- |
|  | **Community dialogue** | | | **Community dialogues** | | | **Women reflection circles** | | | |
|  | **P-value** | **P-wyoung** | **P-bonf** | **P-value** | **P-wyoung** | **P-bonf** | **P-value** | | **P-wyoung** | **P-bonf** |
| Beliefs favourable to cutting | <0.001 | <0.001 | <0.001 | <0.001 | <0.001 | <0.001 | 0.860 | 0.900 | | 0.860 |
| Intends to cut daughter | <0.001 | <0.001 | <0.001 | <0.001 | <0.001 | <0.001 | 0.001 | 0.010 | | 0.002 |
| Agrees that uncut girls are not marriageable | <0.001 | <0.001 | <0.001 | <0.001 | <0.001 | <0.001 | 0.061 | 0.020 | | 0.123 |
| Opposes FGM | <0.001 | <0.001 | <0.001 | <0.001 | <0.001 | <0.001 | 0.039 | 0.010 | | 0.116 |

**Notes:** Values are p-values from survey-weighted logistic regression models, estimated separately for each outcome. All models adjust for the other interventions (community dialogues or women’s circles), marital status, education, age group, ethnicity, and religion among women (sex = 1). “p-value” is the conventional model p-value; “pwyoung” is the Westfall-Young family-wise adjusted p-value controlling for multiple testing across the four outcomes within each platform; “pbonf” is the Bonferroni-adjusted p-value.
