# Supplementary material for: Shifting social norms, behavioural intentions and opposition to female genital mutilation: Effectiveness of layered community interventions in a high prevalence setting in Kenya
Source: PLOS Glob Public Health. 2026 Sep 10;6(9):e0007044. doi: 10.1371/journal.pgph.0007044 (PMC13561337; doi:10.1371/journal.pgph.0007044)
Supplement: Supplementary file 3 [file pgph.0007044.s003.docx]

**S3 Table. Additive effects of women’s reflection circles and community dialogues on FGM norms and opposition among women**

|  | **Probability**  **(%)** | **Probability difference** | **95% CI** | **P-value** |
| --- | --- | --- | --- | --- |
| **Beliefs favourable to cutting** | | | | |
| No intervention | 75.0 | - | - | - |
| Women circle vs none | 74.4 | -0.6 | (-7.7 to - 6.5) | 0.869 |
| Community dialogues vs none | 58.6 | -16.4 | (-21.5 to -11.3) | <0.001 |
| All two | 57.8 | -17.1 | (-25.5 to - 8.7) | <0.001 |
| **Intends to cut daughter** | | | | |
| No intervention | 38.6 | - | - |  |
| Women circle vs none | 24.2 | -14.4 | (-22.0 to -6.8) | <0.001 |
| Community dialogues vs none | 22.5 | -16.0 | (-21.0 to -11.1) | <0.001 |
| All two | 12.7 | -25.9 | (-32.5 to -19.3) | <0.001 |
| **Believes that uncut girls are not marriageable** | | | | |
| No intervention | 49.1 | - | - |  |
| Women circle vs none | 34.3 | -14.8 | (-29.5 to -0.2) | 0.046 |
| Community dialogues vs none | 34.6 | -14.5 | (-21.4 to -7.6) | <0.001 |
| All two | 22.1 | -27.0 | (-39.7 to -14.1) | <0.001 |
| **Opposes FGM** | | | | |
| No intervention | 61.9 | - | - | - |
| Women circle vs none | 73.1 | 11.2 | (1.0 to 21.0) | 0.032 |
| Community dialogues vs none | 78.4 | 16.5 | (12.2 to 20.8) | <0.001 |
| All two | 86.0 | 24.1 | (15.9 to 32.4) | <0.001 |
