# Supplementary material for: Shifting social norms, behavioural intentions and opposition to female genital mutilation: Effectiveness of layered community interventions in a high prevalence setting in Kenya
Source: PLOS Glob Public Health. 2026 Sep 10;6(9):e0007044. doi: 10.1371/journal.pgph.0007044 (PMC13561337; doi:10.1371/journal.pgph.0007044)
Supplement: Supplementary file 4 [file pgph.0007044.s004.docx]

**S4 Table. Internal consistency and reliability of FGM beliefs scale**

| **Item** | **Observations** | **Item-Test Correlation** | **Item-Rest Correlation** | **Alpha** |
| --- | --- | --- | --- | --- |
| Cut girls are healthy | 2,168 | 0.7449 | 0.5534 | 0.6788 |
| Cut girls are clean | 2,151 | 0.7792 | 0.6079 | 0.6569 |
| Cut girls are pure | 1,981 | 0.6950 | 0.4915 | 0.7020 |
| Cut girls are modern | 2,087 | 0.6311 | 0.3974 | 0.7376 |
| Cut girls different* | 2,175 | 0.6707 | 0.4397 | 0.7185 |
| Scale (overall) | — | — | — | 0.7448 |

* Item reverse-coded prior to analysis.
