# Supplementary material for: Shifting social norms, behavioural intentions and opposition to female genital mutilation: Effectiveness of layered community interventions in a high prevalence setting in Kenya
Source: PLOS Glob Public Health. 2026 Sep 10;6(9):e0007044. doi: 10.1371/journal.pgph.0007044 (PMC13561337; doi:10.1371/journal.pgph.0007044)
Supplement: Supplementary file 5 [file pgph.0007044.s005.docx]

**S1 Fig. Pathways from community dialogues to opposing FGM: Survey GSEM results**

**Notes:** Numbers on the arrows are survey-weighted log-odds (logit) coefficients from the GSEM. Green arrows indicate positive coefficients, and red arrows indicate negative coefficients. The arrow from Community Dialogues to agency represents the direct effect (0.47, p = 0.040); the remaining paths correspond to the structural relations whose products form the path-specific indirect effects reported in Table 5. All models adjust for sex, marital status, education level, age category, ethnicity, and religion. Levels of significance: * p < .05, ** p < .01, *** p < .001.
