# Supplementary material for: Shifting social norms, behavioural intentions and opposition to female genital mutilation: Effectiveness of layered community interventions in a high prevalence setting in Kenya
Source: PLOS Glob Public Health. 2026 Sep 10;6(9):e0007044. doi: 10.1371/journal.pgph.0007044 (PMC13561337; doi:10.1371/journal.pgph.0007044)
Supplement: Supplementary file 6 [file pgph.0007044.s007.docx]

**Reviewer response**

**Journal requirements/ Editors comments**

**Comment:** In the ethics statement in the Methods, you have specified that verbal consent was obtained. Please provide additional details regarding how this consent was documented and witnessed, and state whether this was approved by the IRB.

**Response:** Thank you. We have now provided detailed information on how verbal consent/assent was obtained and confirm that the waiver for written consent was approved by the IRB.

**Comment:** Please amend your detailed Financial Disclosure statement. This is published with the article. It must therefore be completed in full sentences and contain the exact wording you wish to be published.

**Response:** Now revised.

**Comment:** Please clarify all sources of financial support for your study. List the grants, grant numbers, and organizations that funded your study, including funding received from your institution. Please note that suppliers of material support, including research materials, should be recognized in the Acknowledgements section rather than in the Financial Disclosure. 
ii. State the initials, alongside each funding source, of each author to receive each grant. For example: "This work was supported by the National Institutes of Health (####### to AM; ###### to CJ) and the National Science Foundation (###### to AM)."

**Response:** Now updated. Thank you.

**Comment:** State what role the funders took in the study. If the funders had no role in your study, please state: “The funders had no role in study design, data collection and analysis, decision to publish, or preparation of the manuscript.”

**Response:** Now updated. Thank you.

**Comment:** If any authors received a salary from any of your funders, please state which authors and which funders.

**Response:** We have now updated the financial disclosure and indicated authors who received a salary from the funder.

**Comment:** Please ensure that your Ethics Statement is available in its entirety at the beginning of your Methods section, under a subheading 'Ethics Statement'.

**Response:** Thank you. We have revised and moved it as advised.

**Comment:** We have noticed that you have uploaded Supporting Information files, but you have not included a list of legends. Please add a full list of legends for your Supporting Information files after the references list.

**Response:** Now added.

**Comment**: In the online submission form, you indicated that “All data relevant to the study are included in the article or uploaded as supplementary information. The data that support the findings of this study are available from the corresponding author upon reasonable request.”. All PLOS journals now require all data underlying the findings described in their manuscript to be freely available to other researchers, either

i. In a public repository,
ii. Within the manuscript itself, or
iii. Uploaded as supplementary information.

This policy applies to all data except where public deposition would breach compliance with the protocol approved by your research ethics board. If your data cannot be made publicly available for ethical or legal reasons (e.g., public availability would compromise patient privacy), please explain your reasons by return email and your exemption request will be escalated to the editor for approval. Your exemption request will be handled independently and will not hold up the peer review process, but will need to be resolved should your manuscript be accepted for publication. One of the Editorial team will then be in touch if there are any issues.

**Response:** Thank you for this important information. We are in the process of publishing the dataset in Dataverse – a public repository that allows access and usage of the data by other researchers. We will provide a link (DOI) to this data once this process is complete. We request at least 30 days to finalize this process.

**Comment:** If the reviewer comments include a recommendation to cite specific previously published works, please review and evaluate these publications to determine whether they are relevant and should be cited. There is no requirement to cite these works unless the editor has indicated otherwise.

**Response:** We thank the editor for this guidance. The reviewer did recommend citing a specific publication (<https://pubmed.ncbi.nlm.nih.gov/37539674/>). We carefully reviewed the suggested article to assess its relevance to the manuscript. However, we determined that its primary focus is on the clinical and health consequences of FGM/C, rather than on community-based interventions, participation dynamics, or methodological issues related to selection bias and causal inference, which are the central concerns raised in the review.

As the manuscript already includes appropriate references on the health implications of FGM (refer to page 5 in the introduction section) and is primarily focused on the evaluation of social norms interventions, we did not consider this additional citation to be essential to the arguments presented. We have therefore not included it, in line with the journal’s guidance that such citations are not mandatory where relevance is limited.

**Reviewers' comments**

**General comment:** This manuscript evaluates the association between community-based interventions (community dialogues and women-only reflection circles) and FGM-related beliefs, intentions, and opposition in Isiolo County, Kenya using cross-sectional survey data from 2,235 respondents. The topic is highly relevant to global public health and gender equity. The study contributes empirical quantitative evidence to an area dominated by qualitative or descriptive research. The analytical approach (GEE models, predicted probabilities, and mediation analysis using GSEM) is interesting and potentially valuable. However, several methodological and reporting issues require clarification before the study can be considered robust. In particular:

i. the causal interpretation of cross-sectional data
ii. selection bias in exposure to interventions construction
iii. and validation of the belief index
iv. interpretation of mediation results clarity in statistical reporting

Overall, the manuscript is promising but would benefit from major revision.

**Response:** We thank the reviewer for this thoughtful and constructive assessment of the manuscript and for recognising both the relevance of the topic and the contribution of the study to the quantitative evidence base on FGM-related social norms. We appreciate the positive feedback on the analytical approach, including the use of GEE models, predicted probabilities, and mediation analysis.

We acknowledge the important methodological and reporting concerns raised, particularly regarding causal interpretation in cross-sectional data, potential selection bias in intervention exposure, the construction and validation of the belief index, and the clarity of mediation analysis and statistical reporting. These are critical issues, and we have carefully revised the manuscript to address each point in detail. A point-by-point response outlining the revisions made is provided below.

**Comment:** Causal interpretation with cross-sectional data

The study relies on cross-sectional data collected over a short time window. Despite acknowledging this limitation, the manuscript frequently uses language implying causal relationships between participation in dialogues and changes in beliefs, intentions, and opposition to FGM. Because exposure and outcomes were measured at the same time, the temporal sequence cannot be established. Individuals who already oppose FGM may be more likely to participate in community dialogues.

**Recommendation:** The authors should: avoid causal wording such as “effect”, “influence”, or “pathways”. Frame findings strictly as associations. Clarify that mediation results reflect theoretical relationships rather than demonstrated causal mechanisms.

**Response:** We thank the reviewer for this important and thoughtful comment. In response,

i. we have carefully revised the manuscript throughout to avoid causal language (e.g., “effect,” “influence,” and “pathways”) and now consistently frame results as associations. Specifically, we have revised the Results and Discussion sections to reflect associative interpretations, including for the analysis of combined exposures and subgroup patterns.
ii. We have also clarified the interpretation of the mediation analysis in both the Methods and Results sections. In the Methods, we now explicitly state that the generalized structural equation model provides a theory-informed decomposition of associations across related variables, rather than evidence of causal mediation (Refer to page 14).
iii. Finally, we have strengthened the limitations section to more clearly acknowledge the constraints of cross-sectional data, including the inability to establish temporal ordering and the potential for selection into intervention exposure.

**Comment:** Selection bias in participation. Exposure to the interventions is based on self-reported attendance at community dialogues or reflection circles. Participation in such programs is likely voluntary. This introduces a strong possibility that individuals attending the programs were already more motivated, more educated, or already opposed to FGM. The manuscript does not address this issue adequately.

**Recommendation:** The authors should: describe the recruitment process for the intervention. Discuss the possibility of self-selection bias. Consider analytical strategies to partially address this issue, such as sensitivity analyses or propensity score approaches.
Discuss and cite <https://pubmed.ncbi.nlm.nih.gov/37539674/>

**Response:** We thank the reviewer for highlighting the possibility of self-selection into intervention participation. We have included more text describing the recruitment process and efforts made to ensure balanced participation. We agree that attendance at community dialogues and reflection circles was voluntary and that respondents who participated may have differed systematically from non-participants in ways related to FGM attitudes and opposition. We have now clarified the recruitment and implementation process in the methods and strengthened the limitations section to note the potential for selection bias and residual confounding. Because the data are cross-sectional and exposure was self-reported, we interpret all findings as adjusted associations rather than causal effects.

We considered propensity-score approaches as a possible sensitivity analysis; however, given the absence of baseline measures and the likelihood of unmeasured differences in motivation and prior attitudes, such methods would only partially address selection concerns. We therefore prioritised transparent interpretation and fuller discussion of this limitation.

We also reviewed the suggested paper. As it focuses primarily on the health consequences of FGM/C rather than intervention participation or evaluation methods, we did not rely on it for the selection-bias discussion, though we have considered it for the broader background on FGM harms.”

**Comment:** Construction of the pro-FGM belief index. The pro-FGM belief index is constructed from five items that are dichotomized and then summed. Respondents endorsing three or more items are classified as holding pro-FGM beliefs. However, the manuscript does not report:

i. internal consistency of the scale validation procedures
ii. justification for the threshold used. Dichotomizing the scale may reduce statistical power and obscure meaningful variation.

**Recommendation:** The authors should: report reliability measures such as Cronbach’s alpha, justify the chosen threshold, consider analysing the index as a continuous variable.

**Response**: Thank you for this important comment. We have substantially strengthened the description and validation of the pro-FGM belief index in the revised manuscript.

First, we now report internal consistency statistics for the five-item scale as shown in the table below. The index demonstrated good reliability (Cronbach’s alpha = 0.74), with item-test correlations ranging from 0.63 to 0.78 and item-rest correlations from 0.40 to 0.61, indicating that all items contribute meaningfully to a common underlying construct. No item deletion improved scale performance. These results are now presented in Supplementary S4 Table.

**S4 Table. Internal consistency and reliability of FGM beliefs scale**

| **Item** | **Observations** | **Item-Test Correlation** | **Item-Rest Correlation** | **Alpha** |
| --- | --- | --- | --- | --- |
| Cut girls are healthy | 2,168 | 0.7449 | 0.5534 | 0.6788 |
| Cut girls are clean | 2,151 | 0.7792 | 0.6079 | 0.6569 |
| Cut girls are pure | 1,981 | 0.6950 | 0.4915 | 0.7020 |
| Cut girls are modern | 2,087 | 0.6311 | 0.3974 | 0.7376 |
| Cut girls different* | 2,175 | 0.6707 | 0.4397 | 0.7185 |
| Scale (overall) | — | — | — | 0.7448 |

* Item reverse-coded prior to analysis.

Second, we clarified the rationale for dichotomising individual items. Endorsement of at least one pro-FGM belief was highly prevalent in the sample (97%), limiting the discriminatory value of single items and motivating the use of a composite index.

Third, we expanded the justification for the threshold used to define pro-FGM beliefs (≥3 items). This threshold captures respondents with a predominantly pro-FGM normative orientation rather than isolated or ambivalent agreement and is supported by the empirical distribution of the scale, which was centred around values of 3-4.

**Comment:** The study uses a multistage cluster sampling design but states that the survey is treated as self-weighting because all individuals had equal probability of selection. This assumption requires clearer justification. In multistage sampling designs, unequal cluster sizes can lead to unequal selection probabilities.

**Recommendation:** The authors should: provide details on the number of clusters and cluster sizes justify the assumption of equal weights clarify whether probability weights were considered.

**Response:** We have clarified the sampling design and justification for treating the survey as self-weighting in the revised manuscript. Specifically, the sampling approach involved (i) random selection of clusters (wards and villages), (ii) systematic selection of a fixed number of households within each cluster, and (iii) random selection of one eligible respondent per household. This design results in approximately equal probabilities of selection across individuals. We acknowledge that unequal cluster sizes in multistage sampling can introduce differential selection probabilities. However, in this study, clusters were broadly comparable in size and the use of fixed sampling fractions within clusters minimised potential imbalance. We have revised the Methods section to explicitly reflect this and to clarify the assumptions underlying the use of equal weights.

**Comment:** Interpretation of demographic results. The results show that women have higher odds of pro-FGM beliefs but do not differ significantly from men in intentions to cut or opposition to FGM. This finding is interesting but not sufficiently discussed. Possible explanations include social norms constraining women’s expression of opposition, generational differences, or decision-making dynamics within households. The discussion should elaborate on this result.

**Response:** Thank you for this insightful comment. In the revised manuscript (refer to pages 28-29 in the discussion section), we have expanded the Discussion section to more fully contextualise this result within the broader social norms and gender literature.

Specifically, we clarify that the observed divergence reflects the distinction between internalised norms, behavioural intentions, and expressed opposition in settings where FGM is sustained by strong collective expectations. We note that women, as primary custodians of cultural practices, may internalise pro-FGM beliefs more strongly through processes of socialisation and intergenerational transmission, even where broader attitudes are beginning to shift.

We also elaborate on the role of household and community decision-making structures. In many high-prevalence contexts, decisions regarding FGM are not made individually but are embedded within extended family and kinship systems involving elders and male relatives. As a result, women’s ability to translate beliefs into intentions or openly expressed opposition may be constrained by limited decision-making power and the risk of social sanctions. We further highlight evidence from social norms research showing that individuals, particularly women, may align their expressed attitudes with perceived community expectations even when private views differ.

In addition, we now discuss the potential role of generational dynamics, noting that older cohorts may retain more entrenched pro-FGM beliefs due to historical socialisation, while changes in intentions and opposition may occur more uniformly across men and women where decisions remain collectively determined.
