# Supplementary material for: Shifting social norms, behavioural intentions and opposition to female genital mutilation: Effectiveness of layered community interventions in a high prevalence setting in Kenya
Source: PLOS Glob Public Health. 2026 Sep 10;6(9):e0007044. doi: 10.1371/journal.pgph.0007044 (PMC13561337; doi:10.1371/journal.pgph.0007044)
Supplement: Peer review history

# Peer review: Decision Letter 0

**DOI:** 10.1371/journal.pgph.0007044.r001

**Authors:** Johanna Pruller

22 Mar 2026

PGPH-D-26-00560

Shifting Social Norms, Behavioural Intentions and Opposition to Female Genital Mutilation: Effectiveness of Layered Community Interventions in a High Prevalence Setting in Kenya

PLOS Global Public Health

Dear Dr. Matanda,

Thank you for submitting your manuscript to PLOS Global Public Health. After careful consideration, we feel that it has merit but does not fully meet PLOS Global Public Health’s publication criteria as it currently stands. Therefore, we invite you to submit a revised version of the manuscript that addresses the points raised during the review process.

==============================

Please note that we have only been able to secure a single reviewer to assess your manuscript. We are issuing a decision on your manuscript at this point to prevent further delays in the evaluation of your manuscript. Please be aware that the editor who handles your revised manuscript might find it necessary to invite additional reviewers to assess this work once the revised manuscript is submitted. However, we will aim to proceed on the basis of this single review if possible.

Could you please revise the manuscript to carefully address the concerns raised?

==============================

Please submit your revised manuscript by May 06 2026 11:59PM. If you will need more time than this to complete your revisions, please reply to this message or contact the journal office at globalpubhealth@plos.org. When you're ready to submit your revision, log on to https://www.editorialmanager.com/pgph/ and select the 'Submissions Needing Revision' folder to locate your manuscript file.

Please include the following items when submitting your revised manuscript:

- A letter that responds to each point raised by the editor and reviewer(s). You should upload this letter as a separate file labeled 'Response to Reviewers'.
- A marked-up copy of your manuscript that highlights changes made to the original version. You should upload this as a separate file labeled 'Revised Manuscript with Track Changes'.
- An unmarked version of your revised paper without tracked changes. You should upload this as a separate file labeled 'Manuscript'.

Guidelines for resubmitting your figure files are available below the reviewer comments at the end of this letter.

We look forward to receiving your revised manuscript.

Kind regards,

Johanna Pruller, Ph.D.

PLOS Staff Editor

PLOS Global Public Health

**Journal Requirements:**

1. In the ethics statement in the Methods, you have specified that verbal consent was obtained. Please provide additional details regarding how this consent was documented and witnessed, and state whether this was approved by the IRB

2. Please amend your detailed Financial Disclosure statement. This is published with the article. It must therefore be completed in full sentences and contain the exact wording you wish to be published.

i. Please clarify all sources of financial support for your study. List the grants, grant numbers, and organizations that funded your study, including funding received from your institution. Please note that suppliers of material support, including research materials, should be recognized in the Acknowledgements section rather than in the Financial Disclosure.

ii. State the initials, alongside each funding source, of each author to receive each grant. For example: "This work was supported by the National Institutes of Health (####### to AM; ###### to CJ) and the National Science Foundation (###### to AM)."

iii. State what role the funders took in the study. If the funders had no role in your study, please state: “The funders had no role in study design, data collection and analysis, decision to publish, or preparation of the manuscript.”

iv. If any authors received a salary from any of your funders, please state which authors and which funders.

3. Please ensure that your Ethics Statement is available in its entirety at the beginning of your Methods section, under a subheading 'Ethics Statement'.

4. We have noticed that you have uploaded Supporting Information files, but you have not included a list of legends. Please add a full list of legends for your Supporting Information files after the references list.

5. In the online submission form, you indicated that “All data relevant to the study are included in the article or uploaded as supplementary information. The data that support the findings of this study are available from the corresponding author upon reasonable request.”.

All PLOS journals now require all data underlying the findings described in their manuscript to be freely available to other researchers, either

1. In a public repository,

2. Within the manuscript itself, or

3. Uploaded as supplementary information.

This policy applies to all data except where public deposition would breach compliance with the protocol approved by your research ethics board. If your data cannot be made publicly available for ethical or legal reasons (e.g., public availability would compromise patient privacy), please explain your reasons by return email and your exemption request will be escalated to the editor for approval. Your exemption request will be handled independently and will not hold up the peer review process, but will need to be resolved should your manuscript be accepted for publication. One of the Editorial team will then be in touch if there are any issues.

If the reviewer comments include a recommendation to cite specific previously published works, please review and evaluate these publications to determine whether they are relevant and should be cited. There is no requirement to cite these works unless the editor has indicated otherwise.

**Additional Editor Comments (if provided):**

[Note: HTML markup is below. Please do not edit.]

Reviewers' comments:

Reviewer's Responses to Questions

**Comments to the Author**

1. Does this manuscript meet PLOS Global Public Health’s publication criteria?

Reviewer #1: Yes

**********

2. Has the statistical analysis been performed appropriately and rigorously?

Reviewer #1: Yes

**********

3. Have the authors made all data underlying the findings in their manuscript fully available (please refer to the Data Availability Statement at the start of the manuscript PDF file)?

The PLOS Data policy

Reviewer #1: Yes

**********

4. Is the manuscript presented in an intelligible fashion and written in standard English?

Reviewer #1: Yes

**********

**Reviewer #1:** This manuscript evaluates the association between community-based interventions (community dialogues and women-only reflection circles) and FGM-related beliefs, intentions, and opposition in Isiolo County, Kenya using cross-sectional survey data from 2,235 respondents.

The topic is highly relevant to global public health and gender equity. The study contributes empirical quantitative evidence to an area dominated by qualitative or descriptive research. The analytical approach (GEE models, predicted probabilities, and mediation analysis using GSEM) is interesting and potentially valuable.

However, several methodological and reporting issues require clarification before the study can be considered robust. In particular:

the causal interpretation of cross-sectional data

selection bias in exposure to interventions

construction and validation of the belief index

interpretation of mediation results

clarity in statistical reporting

Overall, the manuscript is promising but would benefit from major revision.

1. Causal interpretation with cross-sectional data

The study relies on cross-sectional data collected over a short time window. Despite acknowledging this limitation, the manuscript frequently uses language implying causal relationships between participation in dialogues and changes in beliefs, intentions, and opposition to FGM.

Because exposure and outcomes were measured at the same time, the temporal sequence cannot be established. Individuals who already oppose FGM may be more likely to participate in community dialogues.

Recommendation

The authors should:

Avoid causal wording such as “effect”, “influence”, or “pathways”.

Frame findings strictly as associations.

Clarify that mediation results reflect theoretical relationships rather than demonstrated causal mechanisms.

2. Selection bias in participation

Exposure to the interventions is based on self-reported attendance at community dialogues or reflection circles. Participation in such programs is likely voluntary.

This introduces a strong possibility that individuals attending the programs were already more motivated, more educated, or already opposed to FGM.

The manuscript does not address this issue adequately.

Recommendation

The authors should:

Describe the recruitment process for the intervention.

Discuss the possibility of self-selection bias.

Consider analytical strategies to partially address this issue, such as sensitivity analyses or propensity score approaches.ù

Discuss and cite https://pubmed.ncbi.nlm.nih.gov/37539674/

3. Construction of the pro-FGM belief index

The pro-FGM belief index is constructed from five items that are dichotomized and then summed. Respondents endorsing three or more items are classified as holding pro-FGM beliefs.

However, the manuscript does not report:

The study uses a multistage cluster sampling design but states that the survey is treated as self-weighting because all individuals had equal probability of selection.

This assumption requires clearer justification. In multistage sampling designs, unequal cluster sizes can lead to unequal selection probabilities.

Recommendation

The authors should:

provide details on the number of clusters and cluster sizes

justify the assumption of equal weights

clarify whether probability weights were considered.

Interpretation of demographic results

The results show that women have higher odds of pro-FGM beliefs but do not differ significantly from men in intentions to cut or opposition to FGM.

This finding is interesting but not sufficiently discussed. Possible explanations include social norms constraining women’s expression of opposition, generational differences, or decision-making dynamics within households.

The discussion should elaborate on this result.

internal consistency of the scale

validation procedures

justification for the threshold used

Dichotomizing the scale may reduce statistical power and obscure meaningful variation.

Recommendation

The authors should:

report reliability measures such as Cronbach’s alpha

justify the chosen threshold

consider analysing the index as a continuous variable.

**********

what does this mean?). If published, this will include your full peer review and any attached files.

**Do you want your identity to be public for this peer review?**  If you choose “no”, your identity will remain anonymous but your review may still be made public.

For information about this choice, including consent withdrawal, please see our Privacy Policy

Reviewer #1: No

**********

[NOTE: If reviewer comments were submitted as an attachment file, they will be attached to this email and accessible via the submission site. Please log into your account, locate the manuscript record, and check for the action link "View Attachments". If this link does not appear, there are no attachment files.]

# Author comment: Author response to Decision Letter 1

**DOI:** 10.1371/journal.pgph.0007044.r002

22 Apr 2026

Attachment Submitted filename: Response to reviewers 22.04.2026.docx

# Peer review: Decision Letter 1

**DOI:** 10.1371/journal.pgph.0007044.r003

**Authors:** Laura Asher

21 Jul 2026

Shifting Social Norms, Behavioural Intentions and Opposition to Female Genital Mutilation: Effectiveness of Layered Community Interventions in a High Prevalence Setting in Kenya

PGPH-D-26-00560R1

Dear Dr. Matanda,

We are pleased to inform you that your manuscript 'Shifting Social Norms, Behavioural Intentions and Opposition to Female Genital Mutilation: Effectiveness of Layered Community Interventions in a High Prevalence Setting in Kenya' has been provisionally accepted for publication in PLOS Global Public Health.

Before your manuscript can be formally accepted you will need to complete some formatting changes, which you will receive in a follow up email. A member of our team will be in touch with a set of requests.

Please note that your manuscript will not be scheduled for publication until you have made the required changes, so a swift response is appreciated.

IMPORTANT: The editorial review process is now complete. PLOS will only permit corrections to spelling, formatting or significant scientific errors from this point onwards. Requests for major changes, or any which affect the scientific understanding of your work, will cause delays to the publication date of your manuscript.

If your institution or institutions have a press office, please notify them about your upcoming paper to help maximize its impact. If they'll be preparing press materials, please inform our press team as soon as possible -- no later than 48 hours after receiving the formal acceptance. Your manuscript will remain under strict press embargo until 2 pm Eastern Time on the date of publication. For more information, please contact globalpubhealth@plos.org.

Thank you again for supporting Open Access publishing; we are looking forward to publishing your work in PLOS Global Public Health.

Best regards,

Laura Asher

Academic Editor

PLOS Global Public Health

***********************************************************

Reviewer Comments (if any, and for reference):

Reviewer's Responses to Questions

**Comments to the Author**

Reviewer #1: All comments have been addressed

**********

publication criteria?

Reviewer #1: Yes

**********

3. Has the statistical analysis been performed appropriately and rigorously?

Reviewer #1: Yes

**********

4. Have the authors made all data underlying the findings in their manuscript fully available (please refer to the Data Availability Statement at the start of the manuscript PDF file)?

The PLOS Data policy

Reviewer #1: Yes

**********

5. Is the manuscript presented in an intelligible fashion and written in standard English?

Reviewer #1: Yes

**********

Reviewer #1: revised version the authors addressed all the reviewers comments

**********

what does this mean?). If published, this will include your full peer review and any attached files.

**Do you want your identity to be public for this peer review?**  If you choose “no”, your identity will remain anonymous but your review may still be made public.

For information about this choice, including consent withdrawal, please see our Privacy Policy

Reviewer #1: **Yes:** Alessandro Libretti

**********
